# Validity of subjective versus objective quality of life assessment in people with schizophrenia

**DOI:** 10.1186/s12888-014-0365-x

**Published:** 2014-12-24

**Authors:** Karen P Hayhurst, Jennifer A Massie, Graham Dunn, Shôn W Lewis, Richard J Drake

**Affiliations:** Institute of Brain, Behaviour and Mental Health, The University of Manchester, Manchester, M13 9PL UK; Laureate House Mental Health Unit, Manchester Mental Health & Social Care Trust, Wythenshawe Hospital, Southmoor Road, Manchester, UK; Centre for Biostatistics, Institute of Population Health, The University of Manchester, Manchester, UK

**Keywords:** Adherence, Adverse effects, Attitude, Depression, Quality of Life, Schizophrenia

## Abstract

**Background:**

Quality of life (QoL) is considered an important outcome in health research. It can be rated by the patient, or by an external assessor. We wished to identify the predictors of any discrepancies between these two approaches in people with schizophrenia.

**Methods:**

Patients with DSM schizophrenia and related disorders (N = 80) completed both patient-rated (Lancashire Quality of Life Profile; LQOLP) and assessor-rated (Heinrich’s Quality of Life Scale; QLS) measures of QoL.

**Results:**

Patient-rated (LQOLP) and assessor-rated (QLS) measures showed a modest correlation (*r* = 0.38). In a regression analysis, independent predictors of subjectively-rated QoL being higher than objectively-assessed QoL in the same patient, were low insight score (BIS), negative symptoms (PANSS), absence of depression (CDSS), and less positive attitude toward prescribed treatment (DAI).

**Conclusions:**

In people with schizophrenia, scores on objectively- and subjectively-rated measures of quality of life can differ markedly. When comparing subjective to objective assessments, patients with depressive symptoms will value their QoL lower, and those with low insight will value their QoL higher. This has important implications for the utility and interpretation of QoL measures in schizophrenia.

## Background

Quality of life (QoL) is an important outcome in schizophrenia research and was the primary outcome of the UK CUtLASS trials (Cost Utility of the Latest Antipsychotics in Severe Schizophrenia) [1-3]. Self- or patient-rated measures of QoL in schizophrenia can be viewed with suspicion by the clinician [4] due to the perceived impact of depressive and psychotic symptoms, poor insight, and cognitive deficits [5,6]. The alternative is clinician- or assessor-rated measures of QoL but these show only moderate correlation with self-ratings [7-9].

Existing literature converges on extensive differences between the predictors of subjective and objective QoL. Previous researchers have found, for example, with the use of factor analysis, that subjective and objective QoL components cluster separately [10]. The majority of studies have examined the predictors of patient-rated and assessor-rated QoL separately, for example, research on outpatients with schizophrenia concluded that objective and subjective QoL measures have different clinical predictors and should be considered separate outcomes [11-13]. Similarly, a recent review highlighted that few studies had investigated the relationship between subjective and objective QoL assessment, concluding that researchers ought to use both approaches as complementary outcome measures in schizophrenia [14]. This message was re-iterated by Awad and Voruganti in their 2012 update on QoL measurement in schizophrenia [15].

Two major QoL measures used for schizophrenia are the objective QLS (Quality of Life Scale) [16] and the LQOLP (Lancashire Quality Of Life Profile) [17]. The QLS consists of a series of questions regarding common social achievements (see Methods section). The LQOLP consists of a series of questions including two visual analogue scales of subjective life satisfaction (see Methods section).

It is not enough, however, to know that the two types of measure differ. An important issue to examine is what makes them differ. QoL measurement discrepancy serves to highlight fundamental differences between the priorities of the patient and those of the clinician, which may drive patient dissatisfaction with treatment. In addition, knowledge of the drivers of QoL discrepancy can inform research using QoL as an outcome measure.

Despite a large QoL evidence base, few researchers have specifically examined predictors of discrepancy between the two methods of QoL measurement in the same individual. One such study did suggest that patients with QoL measurement concordance were more likely to be young, male and have high symptom scores [18]. Patient ratings in this study, however, were measured using a subjective measure of function; the Subjective Well-being on Neuroleptics (SWN) scale [19], rather than with a specific quality of life measure [18]. Other studies have employed specific QoL measures. Poor insight was linked to greater discrepancy between objective (Standard of Living Interview SOL-I) [20] and subjective (LQOLP) [17] measures of QoL by Doyle and colleagues [6]. Similarly, Whitty et al. [21] suggest that better insight may serve to lower both subjective (WHO Quality of Life Scale Brief Version) [22] and objective QoL (QLS) [16]. A further study using the patient's global QoL assessment and the interviewer's global QoL assessment from the same measure; the LQOLP [17] highlighted an association between greater QoL measurement discrepancy and fewer affective symptoms [7].

During the UK CUtLASS trials [1-3] QoL of participants at one trial site was assessed using the patient-rated LQOLP [17] in addition to the observer-assessed QLS [16]. This provided the ideal opportunity to assess in detail what factors affect each construct and (most importantly) the discrepancy between the two.

## Methods

### Design

The study was conducted in parallel to the CUtLASS trials [1-3], which comprised two randomised controlled trials (RCTs) recruiting from five centres across the UK. The first trial compared FGA (first generation antipsychotic) drugs with non-clozapine SGAs (second generation antipsychotic). The second of the two trials compared SGA drugs, as a class, with clozapine in treatment-resistant patients (poor response to two or more prior antipsychotics).

### Participants

Individuals with DSM schizophrenia, schizoaffective disorder, or delusional disorder and aged 18–65 years, whose treating clinicians were considering a change of antipsychotic due to lack of response, or intolerable side effects, were eligible for the study. The referring clinician discussed the CUtLASS trials with the participant and supplied them with an information sheet. The Trial Support Clinician checked their eligibility from case notes, assigned a study number and visited within 3 days to obtain written consent [3]. Ethical approval was granted by the North West Multi-Centre Research Ethics Committee.

### Procedure and measures

Inclusion criteria for the first trial were at least one month since the first onset of positive psychotic symptoms and psychiatrist electing to change current treatment because of inadequate clinical response or intolerance [1]. Inclusion criteria for the second trial were the responsible clinician electing to change current treatment because of poor clinical response (and considering clozapine), plus trials of at least two previous drugs, with poor clinical response [2]. Exclusion criteria for both trials were substance misuse or a medical disorder considered clinically to be the major cause of positive psychotic symptoms and a history of neuroleptic malignant syndrome [1,2]. This paper reports baseline measures; assessments were carried out by the Trial Support Clinician, a Specialist Registrar in Psychiatry, i.e. ratings of observer-assessed QoL were not made blind to other study ratings.

Participants were assessed using the Positive and Negative Syndrome Scale (PANSS) [23]; the Calgary Depression Scale for Schizophrenia (CDSS) [24]; the Drug Attitude Inventory (DAI) [25]; and the Kemp Adherence Scale [26]. The PANSS [23] is a well-validated measure of symptoms in schizophrenia, providing ratings on three subscales (positive, negative and general psychopathology). The CDSS [24] is widely used as a measure of depressive symptoms in schizophrenia. The DAI [25] and Kemp scale [26] measure attitudes and adherence to treatment.

Treatment-related side effects were measured using the Simpson-Angus scale [27]; the Barnes Akathisia scale [28]; the Assessment of Involuntary Movements Scale (AIMS) [29]; and the Antipsychotic Non-Neurological Side Effects Rating Scale (ANNSERS) [30], a scale with good inter-rater reliability [31] designed to measure adverse events associated with both SGA and FGA treatment.

The primary outcome for the study was quality of life measured on an observer-assessed measure: the Quality of Life Scale (QLS) [16]. The QLS is the most widely used QoL scale in the evaluation of psychopharmacological treatments for schizophrenia [32]. The scale consists of 21 items, with anchored ratings of 0–6, across four domains: social relationships, instrumental role functioning, intra-psychic foundations and activities of daily life. The QLS is rated by trained raters, via a semi-structured interview schedule (e.g. a suggested question for social activity ratings is, "how often have you done things for enjoyment that involve other people?"). It has good inter-rater reliability and confirmatory factor analysis has been carried out. The scale has been shown to be both sensitive to change and of clinical relevance [33]. A measure of high internal consistency (Cronbach α 0.92) was obtained for the 21 items of the QLS assessed at baseline in the CUtLASS study and an inter-rater reliability of 0.99 (intra-class correlation: ICC) achieved for total QLS score; an ICC of 0.84 was obtained for PANSS total score [1]. An initial QLS ICC of 0.91 was obtained for 9 trained raters, using 10 videotaped QLS assessments; further training succeeded in raising the QLS ICC to 0.99 [1].

For the purposes of the current study, 80 participants at the Manchester trial site also completed the widely-used, patient-rated Lancashire Quality Of Life Profile (LQOLP) [17] and the Birchwood Insight Scale (BIS) [34]. The LQOLP was developed out of Lehman’s QoL scale [35] and has both proven reliability [36] and validity [37]. The patient’s global assessment of their own subjective QoL was used for the analysis, which is taken as the average of responses to the general well-being question, completed at the start and end of the LQOLP ("can you tell me how you feel about your life as a whole") [6,38]. The BIS [34] is a widely used self-report measure of insight.

### Data analysis

Scores on each QoL measure were converted into *z* scores, or standard scores (with a mean of zero and unit variance), to allow for an analysis of the statistical determinants of discrepancy between the two measures. Correlations were calculated for *z* score difference between subjective and objective measures (LQOLP minus QLS) and baseline variables. Forwards multiple linear regression analyses of predictor variables (with *p* values of less than 0.2) on patient-rated and assessor-rated QoL and on QoL discrepancy (LQOLP minus QLS) were performed. A *p* value of less than 0.2 was chosen as we did not wish to exclude confounders with trend significance with the potential to affect the relationship between the predictors of interest and the outcome variable. Data were analysed using SPSS for Windows, version 20.

## Results

### Sample

Characteristics of the sample are set out in Table 1. Most patients were male, the median duration of illness was nine years and the sample had a median of three previous hospitalisations.

**Table 1:** **Demographic and clinical characteristics of the sample**

**Baseline variables (N = 80)**			
Gender, Male (%)	54 (68%)		
Smoking, Yes (%)	61 (76%)		
Current drug use, Yes (%)	23 (29%)		
Current alcohol use, Yes (%)	23 (29%)		
	**Mean (SD)**	**Median**	**Range**
Age (yr)	39.8 (10.9)	40.1	18.5 - 60.1
Duration of Illness (yr)	13.16 (11.24)	9.04	0 - 38
Number of previous hospitalisations	4.78 (5.89)	3.00	0 - 40
Patient-rated QoL (LQOLP average)	3.95 (1.48)	3.50	1 – 7
Assessor-rated QoL (QLS total score)	41.61 (17.55)	39.00	11 – 91
Insight (BIS)	12.15 (3.45)	12.00	2 – 16
Depression (CDSS)	9.04 (6.21)	8.00	0 – 22
Positive symptoms (PANSS)	16.89 (6.14)	16.00	7 – 32
Negative symptoms (PANSS)	20.61 (5.49)	20.50	9 – 39
General psychopathology (PANSS)	40.33 (9.74)	40.50	22 - 64
Total symptom score (PANSS)	77.83 (17.44)	76.50	45 – 128
Adherence Rating (Kemp)	5.04 (1.44)	5.00	1 - 7
Drug Attitude (DAI)	+11.08 (10.95)	+10.00	−18 - +30
Simpson-Angus	4.64 (4.23)	4.00	0 – 22
Barnes Akathisia	3.63 (3.18)	3.50	0 – 11
AIMS	1.60 (2.59)	1.00	0 – 15
Non-neurological side effects (ANNSERS)	19.37 (10.13)	20.00	1 – 39
Body Mass Index (BMI)	26.83 (6.33)	26.10	16.5 – 50

At baseline assessment correlation between the two measures of quality of life in those 80 participants with scores on both QoL measures was *r* = 0.384 (*p* = 0.001).

Associations between patient-rated (LQOLP) and assessor-rated (QLS) quality of life and baseline variables were examined (see Table 2).

**Table 2:** **Univariate predictors of patient-rated and assessor-rated quality of life**

	**Patient-Rated QoL (N = 80)**		**Assessor-Rated QoL (N = 80)**	
**Variable**	**Mean**	***p***	**Mean**	***p***
Gender	M = 3.78	0.176	M = 38.28	0.019
	F = 4.27		F = 48.28	
Smoking	Yes = 3.84	0.198	Yes = 38.69	0.007
	No = 4.38		No = 51.59	
Current drug use	Yes = 3.71	0.386	Yes = 37.29	0.185
	No = 4.05		No = 43.30	
Current alcohol use	Yes = 3.63	0.301	Yes = 38.62	0.368
	No = 4.02		No = 42.75	
	***r***	***p***	***r***	***p***
Duration of illness (yr)	0.065	0.588	0.203	0.084
Number of previous hospitalisations	−0.037	0.767	−0.123	0.313
Insight (BIS)	−0.360	0.003	0.136	0.285
Depression (CDSS)	−0.600	<0.001	−0.279	0.016
Positive symptoms (PANSS)	0.011	0.928	−0.285	0.013
Negative symptoms (PANSS)	−0.078	0.511	−0.676	<0.001
General psychopathology (PANSS)	−0.298	0.010	−0.482	<0.000
Total symptom score (PANSS)	−0.190	0.105	−0.590	<0.001
Body Mass Index (BMI)	0.160	0.190	0.180	0.139
Simpson-Angus	−0.048	0.688	−0.066	0.575
Barnes Akathisia	−0.133	0.267	−0.075	0.523
AIMS	0.099	0.410	0.076	0.521
Non-neurological side effects (ANNSERS)	−0.254	0.030	−0.056	0.635
Adherence Rating (Kemp)	−0.217	0.063	0.215	0.065
Drug Attitude (DAI)	−0.145	0.222	0.422	<0.001

In order to determine the factors associated with QoL measurement discrepancy, we firstly examined predictors of the LQOLP and the QLS, separately.

### Patient-rated quality of life

The following variables were entered into a forwards multiple linear regression on the basis of a univariate relationship with a *p* value of less than 0.2 (see Table 2); gender, smoking status, insight (BIS), depression (CDSS), general psychopathology score (PANSS), BMI, ANNSERS score and adherence rating. Table 3 shows that patient-rated (LQOLP) quality of life was statistically determined by depression (CDSS), insight (BIS), non-neurological side effect score (ANNSERS) and adherence rating (Kemp).

**Table 3:** **Statistical determinants of quality of life and of measurement discrepancy**

**Model Predictors**	***B***	**SE**	**Beta**	***p***	**95%** **CI**	**R square change**	**F change**	**Sig. F change**
**QoL discrepancy (patient-rated minus assessor-rated QoL** ***z*** ** score)**								
Insight (BIS)	−0.04	0.04	−0.14	0.243	−0.12 to 0.03	0.204	15.63	<0.001
Negative symptoms (PANSS)	0.09	0.02	0.39	<0.001	0.04 to 0.13	0.097	8.34	0.005
Depression (CDSS)	−0.07	0.02	−0.39	<0.001	−0.11 to −0.03	0.090	8.75	0.004
Drug attitude (DAI)	−0.04	0.01	−0.35	0.003	−0.06 to −0.01	0.084	9.31	0.003
**Patient-rated quality of life**								
Depression (CDSS)	−0.16	0.03	−0.68	<0.001	−0.22 to −0.10	0.339	30.26	<0.001
Insight (BIS)	−0.09	0.04	−0.22	0.029	−0.17 to −0.01	0.056	5.39	0.024
Non-neurological side-effects (ANNSERS)	0.04	0.02	0.28	0.025	0.01 to 0.08	0.040	4.08	0.048
Adherence (Kemp)	−0.24	0.11	−0.22	0.030	−0.45 to −0.02	0.046	4.93	0.030
**Assessor-rated quality of life**								
Negative symptoms (PANSS)	−2.00	0.33	−0.57	<0.001	−2.65 to −1.35	0.427	48.48	<0.001
Drug attitude (DAI)	0.46	0.15	0.29	0.003	0.17 to 0.75	0.077	9.89	0.003

In the model, depression explains 34% of the variance in score on the LQOLP, insight a further 5.6%, non-neurological side effects 4%, and adherence rating a further 4.6% each. These four predictor variables taken together therefore explain 48% of the variance in patient-rated QoL. Increased depression, insight and rated adherence are associated with decreased patient-rated QoL, whereas increased ANNSERS score is associated with increased patient-rated QoL.

### Assessor-rated quality of life

Possible predictor variables were, again, entered into a forwards multiple linear regression on the basis of a univariate relationship with a *p* value of less than 0.2 (see Table 2); gender, smoking status, current drug misuse, duration of illness, depression (CDSS), positive symptoms (PANSS), negative symptoms (PANSS), general psychopathology score (PANSS), BMI, adherence rating and DAI score. Table 3 shows that assessor-rated (QLS) quality of life was predicted by negative symptom score (PANSS) and drug attitude rating (DAI). The main predictor, negative symptom score, explains 43% of the variance in Heinrich’s QLS. A one-point increase in PANSS negative symptom subscale score is associated with a substantial decrease in assessor-rated QoL of two points. DAI score explains a further 7.7% of the variance, with these two variables together explaining 50% of the variance in assessor-rated QoL. Although significant correlations were observed between adherence rating and DAI score (*r* = 0.434, *p* < 0.001) and between PANSS subtotal scores (PANSS positive with PANSS negative *r* = 0.222, *p* < 0.05; PANSS positive with PANSS general *r* = 0.616, *p* = <0.001; PANSS negative with PANSS general *r* = 0.496, *p* < 0.001) multi-collinearity was not present in the dataset (tolerance of above 0.5 and VIF less than 2.0 for each predictor).

### Factors associated with QoL measurement discrepancy

The following variables were entered into a forwards multiple linear regression on the basis of a univariate relationship with a *p* value of less than 0.2 (see Table 4); gender, smoking status, insight (BIS), depression (CDSS), positive symptom score (PANSS), negative symptom score (PANSS), non-neurological side effect (ANNSERS) score, adherence rating (Kemp) and drug attitude (DAI) score. Table 3 shows that the final model contained insight (BIS), negative symptom score (PANSS), depression (CDSS) and drug attitude (DAI) score. Insight explains 20% of the variance in QoL measurement discrepancy, negative symptoms a further 9.7%, depression a further 9% and drug attitude score a further 8.4%. Together these four predictor variables therefore explain 48% of the variance in measured quality of life discrepancy. Relative to objective quality of life, subjective quality of life is scored lower in the presence of greater insight, greater depression, greater concordance and reduced symptoms.

**Table 4:** **Univariate predictors of quality of life measurement discrepancy**

		**Patient-rated minus Assessor-rated QoL** ***z*** **score**	
**Variable**		**Mean**	***p***
Gender	Male	+0.21	0.117
	Female	−0.22	
Smoking	Yes	+0.15	0.153
	No	−0.32	
Current drug use	Yes	+0.21	0.494
	No	+0.00	
Current alcohol use	Yes	+0.19	0.484
	No	−0.02	
**Variable**		***r***	***S***
Length of illness (yr)		−0.152	0.216
Number of previous hospitalisations		0.036	0.779
Insight (BIS)		−0.452	<0.001
Depression (CDSS)		−0.321	0.007
Positive symptoms (PANSS)		0.272	0.023
Negative symptoms (PANSS)		0.432	<0.001
General psychopathology (PANSS)		0.098	0.420
Total symptom score (PANSS)		0.293	0.014
BMI		−0.067	0.595
Simpson-Angus		0.010	0.936
Barnes Akathisia		−0.117	0.338
AIMS		0.034	0.782
ANNSERS		−0.209	0.082
Adherence Rating (Kemp)		−0.374	0.001
Drug Attitude (DAI)		−0.483	<0.001

## Discussion

A significant but moderate correlation (*r* = 0.38) was found between patient-rated and assessor-rated measures of QoL in patients with schizophrenia (N = 80) participating in a clinical trial. This confirms previous findings [7,8,39], whilst pointing to the existence of differing constructs of the concept of QoL on the part of the patient and the assessor, or clinician. Separate analyses of the two QoL measures allowed us to understand the drivers of the discrepancy between the two; a previously under-researched topic.

As in previous studies, for example, Narvaez et al. [11], good insight and depression predicted low patient-rated QoL [7,8,11,12,39-46] but not assessor-rated QoL, driving the discrepancy between the two measures. Previous work has highlighted the relationship between good insight and depression [47]. Increased insight will serve to decrease the patient’s rating of their own QoL, to bring it more in line with the rating given by an observer, thereby reducing QoL measurement discrepancy. Good insight could be toxic, in that it can erode self-esteem and promote self-stigmatisation; but it may also promote a positive, integrated sense of self [48,49]. Of course, rating one’s quality of life as worse is not the same as experiencing a life of poorer quality.

Although QLS and subjective QoL global ratings were correlated, this could conceal substantial variation in the relationship between different aspects of objective QoL and satisfaction. Edmondson and colleagues [50] discovered this when comparing change in various measures of social functioning and satisfaction with them. They argued that this could be explained by Zissi and Barry’s [51] suggestion of a shift in aspirations as function improves.

Negative attitudes to medication, as measured by DAI, predicted lower objective QoL but not self-rated QoL, increasing the discrepancy. Negative attitude toward prescribed treatment is associated by the observer with reduced QoL in a way that patients do not perceive.

Good adherence (based on openly expressed attitudes and medication-related behaviour) predicted low concurrent patient-rated QoL but not assessor-rated QoL nor QoL measurement discrepancy. Other research has suggested that direct links between adherence and QoL are weak or absent [52,53], although this pattern was observed using self-report, rather than externally-assessed, adherence. This type of study is not designed to address the meaning of adherence to individuals and hence understand how it affects QoL [54]. One possibility is that good adherence, if it does not spring from positive attitudes to treatment and a sense of self-efficacy, reflects a sense of burden by illness or services. Positive attitude to medication (rather than broader service relationships) against a complex background of emotive attitudes to services and illness might not be enough.

As in previous studies, greater negative symptomatology predicted lower assessor-rated QoL [5,6,8,11-13,39,40,55] but not patient-rated QoL, suggesting that to a clinician, or assessor, extent of negative symptomatology is seen as the prime driver of quality of life. This corresponds with previous survey research indicating that psychiatrists’ concept of QoL is more illness-orientated [56]. Negative symptoms may have little subjective impact on the patient, whereas they are apparent to an observer, or assessor [8]. Others have argued that negative symptoms linked to the deficit syndrome in schizophrenia can serve to protect the individual's self-esteem via social withdrawal [57], although this perspective would suggest that there ought to be a relationship between negative symptoms and subjective QoL, which was not found here. QLS score is influenced by social function, which is linked to negative symptoms. Hence, negative symptoms will impair one form of QoL measurement without affecting the other; enhancing the discrepancy.

Increased experience of non-neurological side effects was predictive of higher self-reported QoL but of neither assessor-rated QoL nor the discrepancy. Patients with a higher QoL rating may report side effects as more severe; these experiences may have a greater impact on the lives of better-functioning individuals. This relationship was only observed for non-neurological side effect scores, not extrapyramidal side effects (EPS); others have found an association between patient-rated QoL and EPS [58]. A significant positive correlation between ANNSERS score and depression score suggests the experience of non-neurological treatment side effects contributes to depressive symptoms.

The study had a number of limitations. Data were collected during baseline assessments of a RCT; the cross-sectional design therefore means that assumptions of causality in the statistical models must be viewed with caution. In addition, the sample analysed here was comprised of patients entered into a RCT, because of poor clinical response or intolerance, although the trial did have broad inclusion criteria. Although conclusions were derived from a relatively small sample (N = 80) QoL predictive factors highlighted by this study are consistent with the existing evidence base.

In terms of measures used, a number of variables found to be associated with QoL in previous studies, for example, neurocognitive deficits, social support, coping style and anxiety, were not assessed in this sample. It is not known whether the statistical determinants of QoL measurement discrepancy observed here will apply to discrepancies between other patient- and assessor-rated QoL measures. Analysis did include demographic variables previously associated with QoL, such as gender, although other demographic data, which may also be associated with QoL, such as social class and educational level, were not available. In terms of the possible effects of medication, the main outcome paper of the CUtLASS study reported no significant effect of antipsychotic drug class on QoL [1]. The CUtLASS study used a depression scale developed for use specifically in schizophrenia and used measures of QoL with proven reliability and validity. The QLS is argued to be largely determined by the deficit symptoms of schizophrenia [59] leading to the strong relationship between the QLS and negative symptoms reported in previous studies [60]. Potentially, the QLS and negative syndrome overlap, as both are reflective of poor social function. However, we found that items for anhedonia, alogia and blunting were, though conceptually distinct, as predictive of the QLS score.

## Conclusions

Our primary aim was to examine predictors of discrepancy between two widely-used patient-rated and assessor-rated quality of life measures in schizophrenia. There was only moderate correlation between them and their predictors differed. Patient-rated QoL appears mainly to reflect depression, whilst assessor-measured QoL reflects negative symptoms. The largest single predictor of QoL measurement *discrepancy* is insight. This explains 20% of its variance (with less insight associated with better subjective but not objective QoL) but its relationship may be mediated by depression and medication concordance so it is not a significant predictor in a multivariate model. Greater negative symptoms predict discrepancy as high scores predict low objective but not subjective ratings of quality of life.

These findings have relevance to both the clinical and the research setting. In terms of practical management, it appears that the alleviation of depressive symptoms rather than the alleviation of negative symptomatology is more likely to lead to the patient rating their quality of life as improved. For studies using QoL as the primary outcome measure it is worth bearing in mind that the inclusion of patients with low insight scores, low depression scores and high negative symptom scores will serve to increase the degree of discrepancy between patient-rated and assessor-rated QoL.

## Abbreviations

AIMS: Assessment of involuntary movements scale
ANNSERS: Antipsychotic non-neurological side effects rating scale
BIS: Birchwood insight scale
BMI: Body mass index
CDSS: Calgary depression scale for schizophrenia
CGI: Clinical global impression
CUtLASS: Cost utility of the latest antipsychotics in severe schizophrenia
DAI: Drug attitude inventory
DSM: Diagnostic and statistical manual of mental disorders
DUP: Duration of untreated psychosis
EPS: Extrapyramidal side effects
FGA: First generation antipsychotic
LQOLP: Lancashire quality of life profile
PANSS: Positive and negative syndrome scale
QoL: Quality of life
QLS: Heinrich's quality of life scale
RCT: Randomised controlled trial
SGA: Second generation antipsychotic
SOL-I: Standard of Living Interview
SWN: Subjective Well-being on Neuroleptics
TSC: Trial support clinician
